# The geographical configuration of a language area influences linguistic diversity

**DOI:** 10.1371/journal.pone.0217363

**Published:** 2019-06-12

**Authors:** John L. A. Huisman, Asifa Majid, Roeland van Hout

**Affiliations:** 1 Centre for Language Studies, Radboud University, Nijmegen, The Netherlands; 2 International Max Planck Research School, Max Planck Institute for Psycholinguistics, Nijmegen, The Netherlands; 3 Department of Psychology, University of York, Heslington, York, United Kingdom; University of Edinburgh, UNITED KINGDOM

## Abstract

Like the transfer of genetic variation through gene flow, language changes constantly as a result of its use in human interaction. Contact between speakers is most likely to happen when they are close in space, time, and social setting. Here, we investigated the role of geographical configuration in this process by studying linguistic diversity in Japan, which comprises a large connected mainland (less isolation, more potential contact) and smaller island clusters of the Ryukyuan archipelago (more isolation, less potential contact). We quantified linguistic diversity using dialectometric methods, and performed regression analyses to assess the extent to which distance in space and time predict contemporary linguistic diversity. We found that language diversity in general increases as geographic distance increases and as time passes—as with biodiversity. Moreover, we found that (I) for mainland languages, linguistic diversity is most strongly related to geographic distance—a so-called isolation-by-distance pattern, and that (II) for island languages, linguistic diversity reflects the time since varieties separated and diverged—an isolation-by-colonisation pattern. Together, these results confirm previous findings that (linguistic) diversity is shaped by distance, but also goes beyond this by demonstrating the critical role of geographic configuration.

## Introduction

The diversity found across the world’s languages today is not the same as it was a hundred or 10,000 years ago, nor will it stay the same in the future. As the processes of diversification need time to run their course, we often find more diversity in areas where a language has been used for longer—compare, for example, English in the United Kingdom with English in Australia [1]. On top of this temporal dimension, we also see that linguistic diversity increases over geographical distance. Several patterns of linguistic diversity have been shown to exist, ranging from gradually accumulating differences [2], to more burst-like diversification [3]. The specific role that the geographical configuration of a language area plays in this process is less explored. The current study aims to investigate to what extent a cultural process such as language diversification follows the same patterns as a biological diversification. To do this, we investigate patterns of linguistic diversity in the context of an island setting by applying insights from population genetics.

There are two notions from population genetics that we investigate in detail here. First we consider dispersal, which is defined as any movement that has the potential to affect gene flow, i.e. the transfer of genes between populations [4]. If dispersal can occur without restriction, genes are transferred across all populations and we find evenly-spread genetic variation and high levels of homogeneity [5]. However, the physical characteristics of the individual put a limit on its dispersal range and this reduces gene flow between distant populations. With this reduced gene flow, genetic differentiation between populations will increase and the end result is increased diversification over geographic distance; a pattern that has been dubbed isolation-by-distance [6].

The same idea can be applied to language. Speakers adapt their speech patterns to accommodate to their most common conversational partners, their speech community [7]. The use of language in human interaction can be thought of as linguistic gene flow. This interaction will, for logistical reasons, be more intense between people that are close to each other: linguistic features first spread across communities that share dense interaction, and then expand into the rest of a language area—a process called diffusion ([8], for an overview). As a result, the language of neighbouring communities will differ only slightly [9]. However, contact between geographically distant communities will be less frequent and accommodation will occur to a lesser degree. This limited linguistic gene flow over increasing geographic distance means that speech communities will resemble each other less and less the farther apart they are [2]—the isolation-by-distance pattern described above. Linguists often call this a dialect continuum and it has been shown to hold over several language areas. Nerbonne [10] investigated language varieties in six areas (Bantu, Bulgaria, Germany, US East Coast, the Netherlands, and Norway), and found linguistic diversity increased over geographic distance.

Although compelling in some ways, the areas investigated to date have focused on land-connected language areas (cf., [11]). It is unclear whether the same generalizations hold for island languages as other factors play a role there. Linguistic dispersal, i.e. contact, requires travel and travel across connected land can, in principle, be done on foot. This lowers the threshold for contact between neighbouring communities, making it easier to maintain connections over longer periods of time. In contrast, travel across islands requires seafaring technology and this limits the amount of contact between island communities.

As such, a second issue to consider is colonisation history [12]. From population genetics, we know that when a new population is started by a small subgroup of a larger one, it will only represent part of the overall diversity found in the original population—known as the founder effect [13]. In isolation, the new population undergoes local genetic adaptation and in time, this leads to a significant divergence from the original population. This divergence reduces the chances of successful colonisation by later waves of migrants from the original population [14]. As such, the diversity we find reflects the time that has passed since the two populations separated and diverged, a pattern that is called isolation-by-colonisation [12].

Similarly, for language, when subgroups of speakers expand into new territory, isolation caused by large distances between island communities has been shown to increase language diversification after settlement [15]. We find that languages diverge in pulses that coincide with each wave of colonisation [16]. While islands have been argued to require wider resource networks due to a greater ecological risk [16]—which would increase contact and in turn decrease linguistic diversity—Lee and Hasegawa [17] show that the presence of a body of water acts as a barrier that promotes diversification. Sustained contact between communities will depend on the distance between islands [18].

The two factors involved in diversification discussed above (dispersal and colonisation history) result in predictable patterns of genetic diversity (isolation-by-distance and isolation-by-colonisation; [12]). Moreover, these factors have been shown to play different roles in specific geographic configurations [19]. Fragmented landscapes, such as archipelagos, have been considered a good setting to investigate how genetic diversity is influenced by geography [20]. Therefore, if the same processes apply to language, as has been argued above, we should be able to make predictions about patterns of linguistic diversity too. To test this, we investigated linguistic diversity in Japan.

The Japanese archipelago is an arc of islands stretching over 2,500 kilometres and comprising over 400 contemporary inhabited islands. Approximately 70% of the land area consists of forested mountains. Ecological risk seems to be low across islands (cf., [16]). Their climate provides self-sufficiency through abundant food sources [21], which is further evidenced by the relatively late arrival of agriculture to the archipelago, despite it being inhabited for a long time [22]. The switch to agriculture happened even later in the southern islands, showing that the survival of its first settlers was supported by the resources available and did not require broader social networks beyond the scope of the island on which they lived.

Spoken across the archipelago is the Japonic language family. Japonic has not been convincingly linked to other languages or language families, but a distant connection to Koreanic seems plausible [23,24]. The language family consists of two main branches: (I) Japanese, which can be subdivided into Eastern, Western, Kyūshū and Hachijō Japanese; and (II) Ryukyuan, which can be subdivided into Amami and Okinawa (Northern Ryukyuan) on one hand, and Miyako, Yaeyama and Yonaguni (Southern Ryukyuan) on the other [25,26]. Both traditional dialectology and computational approaches have shown a clear split between Japanese and Ryukyuan based on the shared presence of Standard Japanese forms [27], the shared presence of linguistic innovations [28], and phylogenetic analyses based on shared cognacy of basic vocabulary [29]. The split is corroborated by politico-cultural history [22], and population structure studies [30,31]. Importantly, Japanese is spoken on the large islands that are close to each other, whereas Ryukyuan is spoken across a number of small island clusters that have relatively large distances between them. We investigated whether these specific geographic configurations influence patterns of linguistic diversity. In addition to Japonic, varieties of Ainu have traditionally been spoken by a distinct indigenous non-Japonic group in the northern parts of Japan. Ainu is critically endangered with few speakers remaining. However, we do not consider Ainu in the current investigation.

While dispersal and colonisation history are both expected to influence language diversification in Japanese and Ryukyuan, we predict that they do so to different degrees. Owing to the relative ease of travel across connected land, dispersal—contact between speakers—is less restricted by natural barriers across the Japanese language area and therefore, gene flow—accommodation between speakers—can occur more freely. As such, we predict that linguistic diversity in Japanese will mostly be a reflection of the distance that speakers can travel: an isolation-by-distance pattern. In contrast, the technological requirements of sea travel limit contact and accommodation across the Ryukyuan language area and local diversification will occur to a larger degree. Therefore, we predict that linguistic diversity in Ryukyuan will mostly reflect the time since language varieties diverged: an isolation-by-colonisation pattern.

## Methods

### Linguistic data

We created a new comparative dataset based on the 100-item Swadesh List ([32]; see also Table 1)—a list of what are considered to be basic concepts, such as body parts and everyday actions. The Swadesh List is well-established in both large-scale and small-scale comparative studies [33,34]. In light of recent findings that the lexicon may be more stable over time than grammatical features [35], we take this list of basic concepts to be a good starting point for comparison. We built on the database collated by Lee and Hasegawa [29], like them using the six-volume *Dictionary of Contemporary Japanese Dialects* [36], but additionally coding the data to preserve all distinctions present in the original material (unlike Lee and Hasegawa, see their Data Supplement 2). Furthermore, we include an additional 11 (mostly island) varieties over the original Lee and Hasegawa database. In addition, we collated data from Volumes 1–3 and 7 of *The Complete Works of Tōsō Miyara* [37], to add another 22 Ryukyuan varieties. Miyara was a Ryukyu-born phonetician, and speaker of one of the local varieties, whose works have been used as a reliable source of contemporary variation, e.g., for the reconstruction of Proto-Ryukyuan [38]. Due to incomplete source material, the eventual dataset contained data for 98 out of the 100 Swadesh List items (Table 1). The data set is available through an Open Science Framework (OSF) archive at https://osf.io/8cxry/. In total, 58 Japanese and 32 Ryukyuan varieties are represented in the data set—see S1 Supporting information for a map of location names).

**Table 1:** Items of the 100-item Swadesh List.

all	full	new	to die
ash	to give	night	to drink
bark	good	nose	to eat
belly	green	not	to kill
big	hair	one	to know
bird	hand	path, road	to lie down
black	head	person	to say
blood	to hear	rain	to see
bone	heart	red	to sit
breasts	horn	root	to sleep
claw	hot	round	to stand
cloud	I	sand	to swim
cold	knee	seed	to walk
dog	leaf	skin	tongue
dry	liver	small	tooth
ear	long	smoke	tree
earth, soil	louse	star	two
egg	man	stone	water
eye	many	sun	we
fat, grease	meat, flesh	tail	what?*
feather	moon	that	white
fire	mountain	this	who?
fish	mouth	to bite	woman
to fly	name	to burn*	yellow
foot	neck	to come	you

Items marked with an asterisk were omitted from this study due to a lack of data.

### Linguistic diversity

Various methods of quantifying linguistic distance have been used in previous research. One approach has been to compare varieties to one “standard”, and calculate distances accordingly [27]. However, comparing to one standard variety does not reveal how different non-standard varieties are from each other, which is important as these non-standard varieties can differ in both the linguistic features they retain, as well as the innovations they pick up. Another approach is to focus on a number of language-specific innovations, e.g., examining vowel shifts or voicing patterns characteristic of one language area [28]. However, this requires both an in-depth knowledge of the language varieties that are being studied, and it limits the number of features that can be compared in a single analysis. Finally, phylogenetic approaches applied to language data require cognate-coding [17,29], which in turn require broad linguistic judgements, and critically reduce the amount of data as non-cognate forms are excluded.

Instead, we adopted a measure of linguistic distance commonly used in dialectometry, based on edit distance—specifically Levenshtein distance [39]. The Levenshtein distance between two strings (e.g., dialect word forms) is calculated as the minimum number of single-character edits needed to turn one into the other. Edits can entail any combination of character additions, deletions, or substitutions. This method was first used in the study of Irish dialects [40] and is a novel approach to analysing linguistic diversity in the Japonic language family. We used Gabmap [41], a free online tool for dialect analysis, to perform the calculations. Gabmap normalises edit distance based on the length of the word forms to take into account the differential impact edits have on short versus long items. Linguistic distance between two locations is then calculated by aggregating Levenshtein distance over a large number of items, an approach that finds its roots in the works of Séguy [42,43] and Goebl [44]. Gabmap also allows for multiple entries per item.

We opted to use the software’s algorithm that assigns linguistically informed costs to the edits involved. In this approach, to preserve syllable, structure substituting a vowel with a consonant, or vice versa, receives double weight. Furthermore, diacritic marks—used to indicate smaller degrees of modification like devoicing or aspiration—are counted as half an edit as they are seen as a smaller deviation from the character they modify than a completely different character would entail. Vowel-consonant substitutes are rare in Japonic varieties given their rigid CV mora structure. While syllabic (moraic) fricatives do occur in Miyako Ryukyuan, e.g. in the Ogami dialect [28], the source material used for the varieties in this study’s dataset did not include such cases. However, diacritic changes are not uncommon. For example, the underlying phonological contrast of front versus back high vowels is maintained across both Tokyo Japanese and the Tohoku dialects, but the phonetic realisation of these vowel in Tohoku is more central, so this is represented as a change in diacritics rather than as a change in characters, coded as /i/ vs. /ï/ and /ɯ/ versus /$\ddot{ɯ}$/. Another example is devoicing of the vowel in the first mora, which is common in some Yaeyama varieties, as found in e.g., *pḁna* ‘nose’ in Hateruma. This is a small, non-phonemic, modification when considering *pana* in Yoron (Amami). However in comparison to *hana* ‘nose’ in Tokyo Japanese there is a change of the initial consonant that is phonemic, and is represented by a character change.

Calculating aggregate distances over all items for all locations within a dataset creates a location-by-location linguistic distance matrix. The method has a number of advantages over previous approaches. It can: (I) make direct comparisons between all varieties of interest, (II) compare all segments in all words, increasing the number of data-points and expanding the comparison beyond specific predetermined items of interest [45], and (III) analyse linguistic data based on surface forms without the need for additional linguistic coding and judgements that potentially decrease the amount of data considered. Finally (IV), it has the additional advantage of examining diversity within a language, rather than merely counting the number of separate languages (cf., [11]).

### Colonisation history

The time-depth and phylogeny of a language family reflects its colonisation history [46] and as such, we used that as a basis to code a *time since divergence* variable. Lee and Hasegawa [29] estimated the time-depth of the Japonic language family in years before present (YBP) using Bayesian phylogenetic analyses. For this, they collated basic vocabulary data for a number of contemporary varieties, and for two older forms of the language (Old Japanese and Middle Japanese). They calibrated the age ranges of Old Japanese (1216–1300 YBP) and Middle Japanese (437–674 YBP), as well as the divergence of the Kyoto and Tokyo varieties (the historical and current capitals, respectively; dated 142–549 YBP), and then constructed a phylogeny of the Japonic language family based on a model incorporating varying rates of linguistic evolution. They found a median age for the split between Japanese and Ryukyuan of 2182 years before present. Using Lee and Hasegawa’s maximum clade credibility tree, we determined the approximate age of the most recent common ancestor (MRCA) for each pair of language varieties, but we generalised time since divergence over all varieties within major subgroups that diverged before 250 YBP. This date was chosen because at this time point, all generally accepted subdivisions in both Japanese (Eastern, Western, Kyushu) and Ryukyuan (Amami, Okinawa, Miyako, Yaeyama, Yonaguni) are represented in the tree.

Within these subgroups, pairwise time since divergence was defined as 50 years younger than the age of the subgroup to which language varieties belonged. This allowed us to include the additional language varieties missing in Lee and Hasegawa’s tree with minimal additional assumptions—particularly in the Ryukyuan language area. For example, the MRCA for Amami and Okinawa in Lee and Hasegawa’s tree was dated at approximately 400 YBP, but since their data set only included one variety of each, we dated the MRCA for the Okinawa varieties in our dataset at 400–50 = 350 YBP. We did not adopt a more fine-grained coding as more recent, relatively small divergences were not expected to have a substantial impact on the outcome since the older divergence between major groups occurred much longer ago—see also the last paragraph in Analysis section below. Importantly, this coding scheme takes the time-depth of larger subgroupings within the two language areas into account, which can be important as language diversity in general increases over time [47]. Time since divergence was coded in a location-by-location matrix.

### Geographic distance

All locations included in the linguistic data were marked in a KML map file using Google Earth. The geospatial data from their coordinates was used to calculate straight-line geographic distances, which were entered into a location-by-location distance matrix. As language distance decay has been shown to be sublinear [45], we created a second distance matrix by performing a natural logarithmic transformation on straight-line geographic distance.

### Separation by water

As the presence of an oceanic barrier has been shown to influence language diversification, we coded a *separation by water* variable for each pair of locations, with value “1” if a body of water separates the two, and with value “0” if not, following Lee and Hasegawa [17]. As our dataset includes a range of both water and land distances, we included this variable to be able to look at the effect of separation by water individually, and along with geographic distance. The binary values were coded as a location-by-location matrix.

### Analysis

We began by verifying the commonly accepted subgroupings of Japanese and Ryukyuan within our data. For this, we analysed the linguistic distance matrix of the Swadesh List data using a hierarchical clustering algorithm based on Ward’s method [48], in R (hclust function, [49]).

Next, we tested to what extent the factors discussed above (geographic distance, time since divergence, and separation by water) are related to linguistic distance. Because we expected the effect of geographic distance to differ between island versus mainland languages [18], we also included an interaction between geographic distance and separation by water in our analyses. Using Mantel tests (ecodist package; [50]), we correlated the four factors with each other to test their relatedness, and then correlated linguistic distance with those same four factors, using partial Mantel tests to control for their mutual influence. All Mantel tests were carried out using 10,000 permutations and 1,000 bootstrap iterations on 95% confidence intervals. To further model linguistic diversification, we performed multiple regression over distances matrices (MRM function, ecodist package; [50]), using the four factors as independent variables and linguistic distance as the dependent variable.

However, MRM analysis has limitations in that it cannot include random effects. We therefore performed an additional linear mixed model analysis on the full distance matrices (lme4 package; [51]) to predict linguistic diversification using the same four variables as before, while adding random intercepts for language varieties to account for their inherent uniqueness. For all mixed models, we will report standardised coefficients (reghelper package; [52]), and include estimates of *p*-values (lmerTest package; [53]), as well as pseudo-R^2^ values (piecewiseSEM package; [54]).

A preliminary analysis of the Japonic language family as a whole (see S1 Appendix) showed that time since divergence was the most important factor across all Mantel and regression analyses. However, the correlation between time since divergence and a binary coded Japanese-vs-Ryukyuan—in which a comparison between one Japanese and one Ryukyuan variety was coded as “1”, and a comparison between two Japanese varieties or two Ryukyuan varieties was coded “0”—was *r* = .980, indicating that the time since divergence variable for all of Japonic primarily represents the split between Japanese and Ryukyuan. All R scripts can be found at the aforementioned OSF archive: https://osf.io/8cxry/.

## Results

### Japanese and Ryukyuan form distinct subgroupings

**Fig 1:**
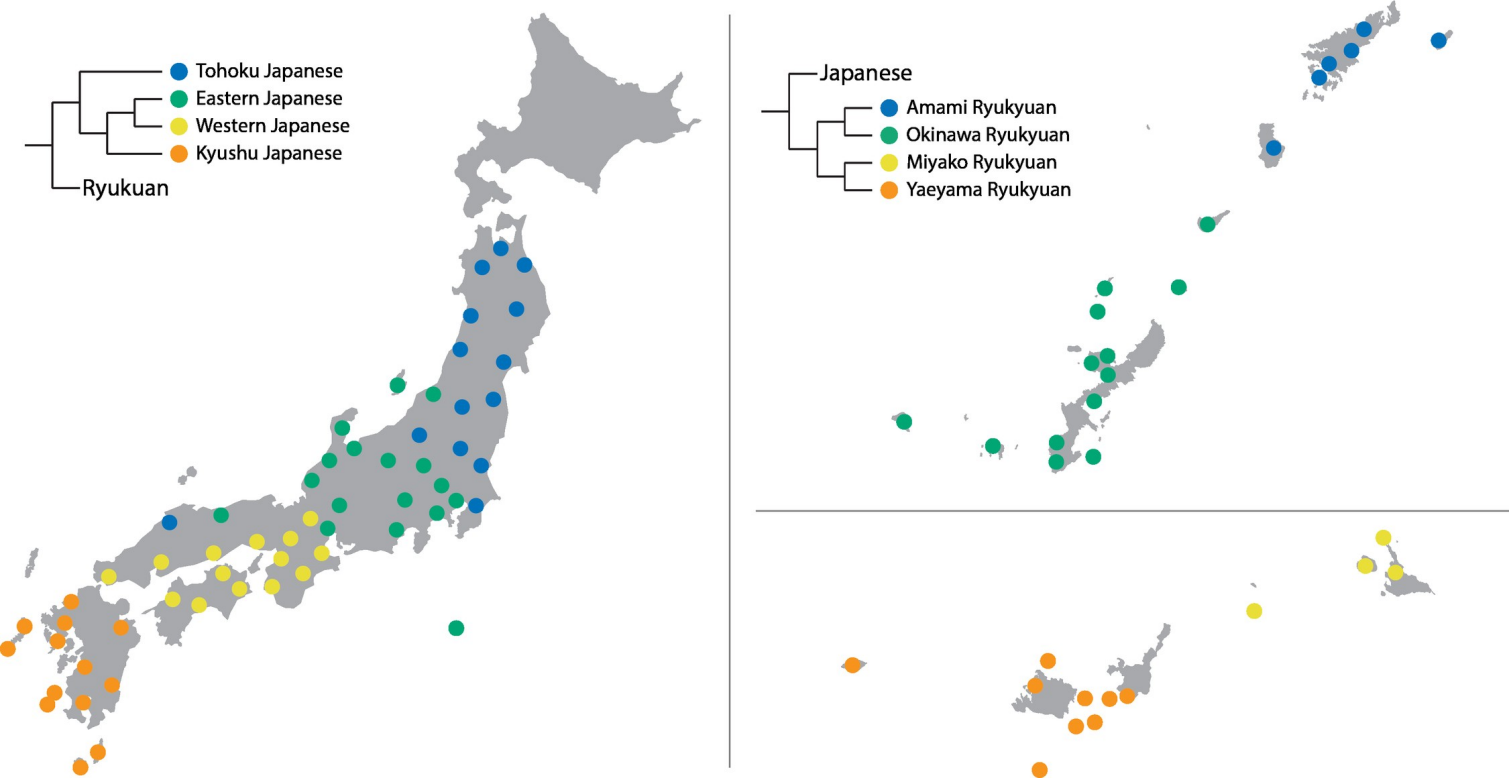
Cluster analysis results for Japanese and Ryukyuan.

The results of the cluster analysis (Fig 1) are in line with both traditional classification in Japanese dialectology [25], and with Lee and Hasegawa’s phylogenetic tree [29]. Critically, the cluster analyses confirmed that Japanese and Ryukyuan are distinct, showing a clear split between all Japanese and all Ryukyuan varieties, replicating previous findings [26]. Discussing all the specific subgroups is unfortunately beyond the scope of this paper. However, for Japanese (Fig 1, left panel) it is noteworthy that while the cluster analysis confirmed the accepted main division between Eastern and Western Japanese varieties, both the peripheral varieties in the north (Tohoku Japanese) and those in the south (Kyushu Japanese) formed distinct subgroups. For Ryukyuan (Fig 1, right panel) the cluster analysis confirmed a main division between Northern Ryukyuan (Amami and Okinawa), and Southern Ryukyuan (Miyako and Yaeyama).

**Fig 2:**
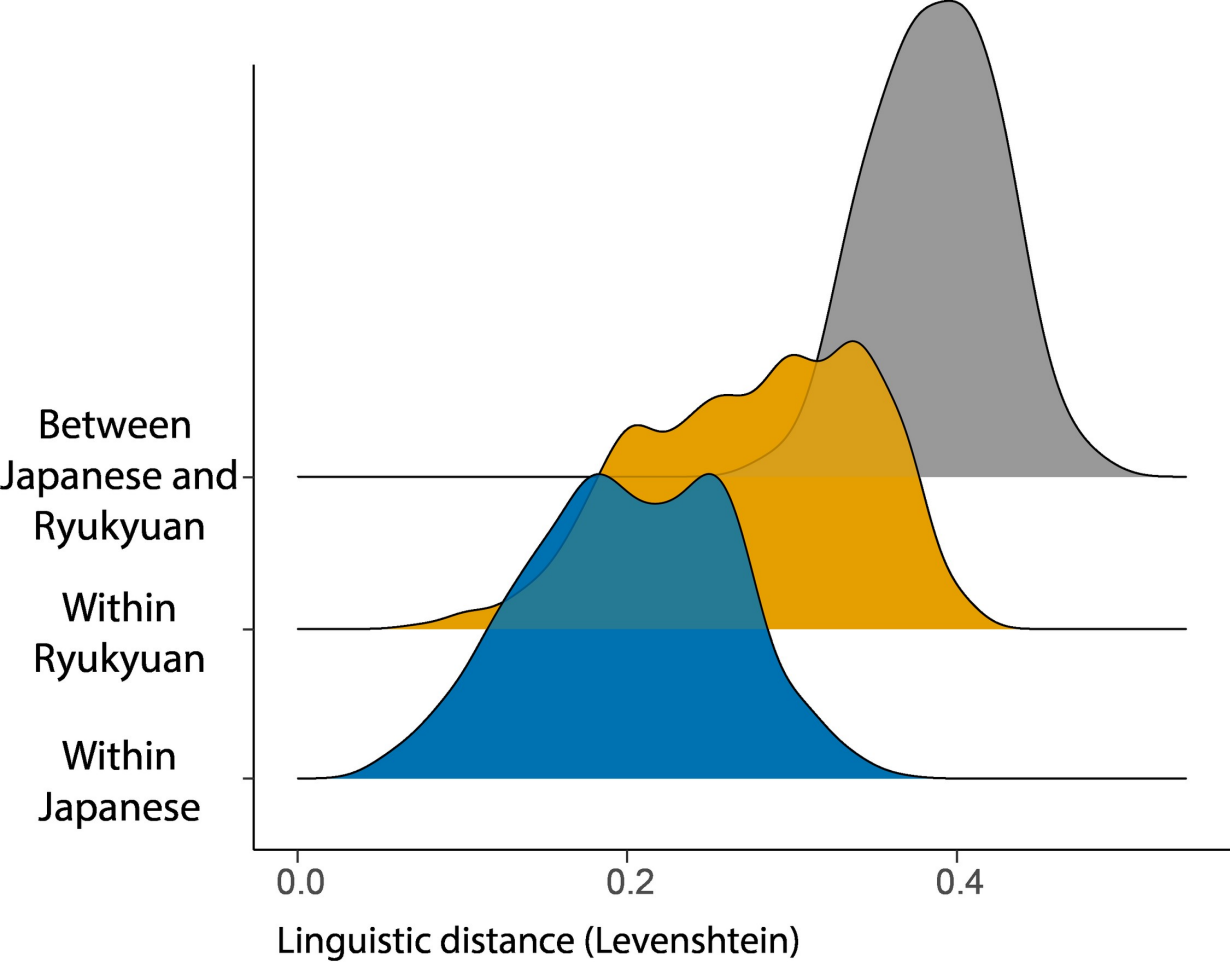
Linguistic distances within Japanese (blue), within Ryukyuan (orange) and between the two language areas (grey).

Fig 2 shows the distribution of linguistic distances within and between Japanese and Ryukyuan. The Japanese distances (blue) show a bimodal distribution, were the second peak corresponds to the large differences between the two peripheral subgroups, Kyushu Japanese and Tohoku Japanese. For Ryukyuan (orange), we see a quadrimodal distribution that corresponds to the four subgroups (Amami, Okinawa, Miyako, and Yaeyama). The four separate modes show that linguistic distances between the subgroups is large, i.e. these subgroups are pronounced in their distinctiveness. Average linguistic distance within Ryukyuan (*M*_*Ryu*_ = 0.256, *SD* = 0.068) was significantly larger than the distance within Japanese (*M*_*Jap*_ = 0.205, *SD* = 0.061), *t*(751.1) = 14.88, *p* < .001, Cohen’s *d* = 0.78. Linguistic distances between the Japanese language area and the Ryukyuan language area (grey) were larger overall and showed a normal-like distribution, indicating that there are no Japanese-Ryukyuan subgroups between which linguistic distances were small.

**Fig 3:**
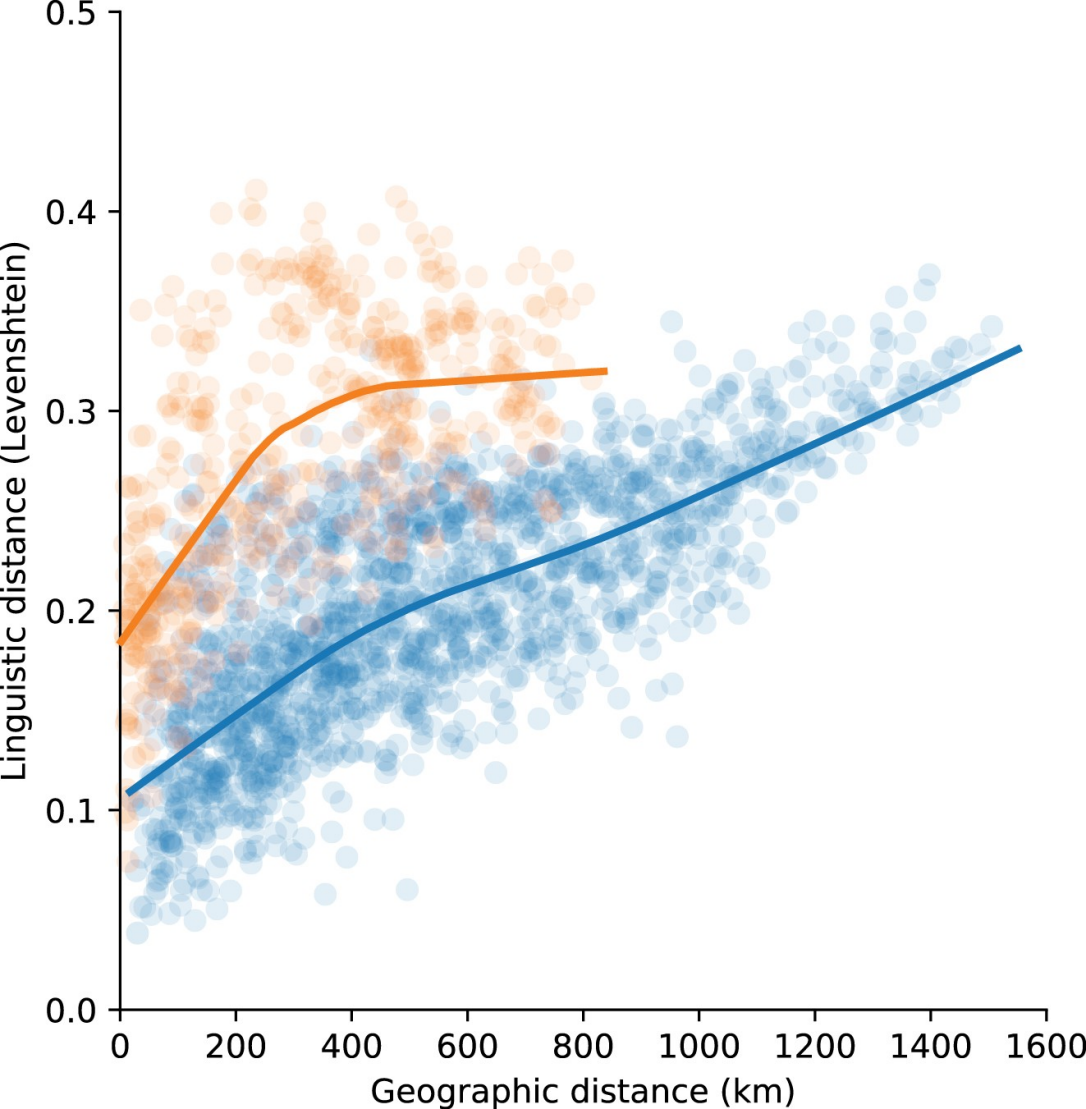
Linguistic distance over geographic distance in Japanese (blue) and Ryukyuan (orange) with Loess smoothing.

Fig 3 shows the distribution of linguistic distances along geographic distance for Japanese (blue) and Ryukyuan (orange), together with a Loess smoothing curve. As described above, linguistic distance in Ryukyuan are larger than in Japanese—despite occurring over smaller geographic distance. In addition, Ryukyuan shows a sharp increase that tapers off quickly, while Japanese showed a moderate increase that continues linearly. This points to Japanese as being more continuum-like where linguistic differences slowly accumulate over geographic distance, which is evidence for an isolation-by-distance pattern. The initial increase in linguistic distance for Ryukyuan shows that this language area also shows continuum-like characteristics on the small scale, but the fact that this levels off fairly quickly shows that beyond a certain point—i.e., beyond the island cluster—linguistic differences are large in genera without a clear connection to geographic distance, evidence for an isolation-by-colonisation pattern.

### Geography and linguistic diversity across the Japanese mainland

Mantel tests confirmed that geographic distance, time since divergence, and separation by water are related to each other across the Japanese mainland (Table 2). Partial Mantel tests then showed that geographic distance was strongly correlated with linguistic diversity (Table 3): linguistic distance between language varieties increased with increased geographic distance. Contrary to what has been previously reported [45], there was no significant difference between linear geographic distance and logarithmic geographic distance in the strength of their association with linguistic distance, *z* = 0.53, *p* = .596. In fact, the correlation with linear distance was numerically higher (*r* = .545 versus *r* = .532). There was no significant correlation between linguistic diversity and time since divergence, nor between linguistic diversity and separation by water for the Japanese varieties. The interaction between geographic distance and separation by water was significant, however, and its negative value indicates that the effect of geographic distance was smaller for varieties separated by a body of water. These findings were supported by the MRM analysis, which confirmed that geographic distance was a significant predictor of linguistic distance, as was the interaction between geographic distance and separation by water, in a model that accounted for 58% of the variation (Table 4).

**Table 2:** Simple Mantel correlations between time since divergence, geographic distance and separation by water for Japanese.

	Time since divergence	Separation by water
Geographic distance	.501	.452
Separation by water	.060	

**Table 3:** Partial Mantel correlations between linguistic distance, time since divergence, geographic distance and separation by water for Japanese.

	Linguistic distance			
	r	95% CI		p
Time since divergence	-.097	-.160	-.040	.129
Geographic distance	.549	.504	.598	< .001
Separation by water	-.001	-.049	.054	.999
Geographic * Water	-.097	-.158	-.058	.041

**Table 4:** Results for predicting linguistic distance in Japanese using multiple regression over distances matrices.

	Estimate	p
Intercept	0.146	
Time since divergence	-8.52·10^−5^	.119
Geographic distance	1.76·10^−4^	< .001
Separation by water	-1.54·10^−5^	.999
Geographic * Water	-2.93·10^−5^	.037

R^2^ = .579.

Coefficients produced by the mixed model analysis (Table 5) were largely in line with results from the Mantel tests, except that all predictors turned out significant in the analysis after including random effects for language varieties. VIF values for the main effects were all < 2.0. The model confirmed the strongest predictor of linguistic distance across the Japanese mainland to be geographic distance—once again, linear geographic distance (AIC = -12648.7) provided a better model than logarithmic distance (AIC = -12443.6). This geographic distance effect was weaker for varieties separated by a body of water. In line with Nettle’s proposal that the increased ecological risk of islands calls for wider social networks [47]—more contact and accommodation, and thus less diversity—varieties separated by water exhibited smaller linguistic distance. The effect of time since divergence, while significant, is much weaker than that of geographic distance. In fact, the negative coefficient indicates that varieties that diverged longer ago are *more* similar to each other, which is a sign that sustained contact (through geographic proximity) can negate the effects of previous isolation. Taken together, these findings show a strong effect of geographic distance on linguistic distance, which confirms our hypothesis that the patterns of linguistic diversity on the mainland should largely reflect contact between speech communities, as predicted by isolation-by-distance.

**Table 5:** Results for predicting linguistic distance in Japanese using linear mixed effect modeling.

	*β*	*SE*	*t*	*p*
(Intercept)	.046	.045	1.02	
Time since divergence	-.040	.013	3.14	< .001
Geographic distance	.809	.016	51.54	< .001
Separation by water	-.111	.014	7.88	< .001
Geographic * Water	-.101	.013	7.87	< .001

Conditional R^2^ = .667, Marginal R^2^ = .551.

### Geography and linguistic diversity across the Ryukyu Islands

Mantel tests confirmed that the predicting factors of linguistic distance are correlated for Ryukyuan as well (Table 6). The partial Mantel tests showed that only the correlation between linguistic distance and time since divergence was significant (Table 7). The longer ago two varieties diverged from each other, the more linguistic distance there was between them. Geographic distance failed to reach significance, and logarithmic geographic distance showed no difference in its correlation with linguistic distance when compared with linear distance, *z* = 0.05, *p* = .960. Moreover, there was little numerical difference between the two; *r* = .067 versus *r* = .064. In contrast with the findings by Lee and Hasegawa [17], separation by a body water did not lead to increased linguistic distance, which can be attributed to the fact that Ryukyuan is spoken on island clusters and the presence of a body of water is not a defining characteristic. Finally, the interaction effect indicated that the influence of geographic distance decreased when language varieties are separated by water, but it was not of significant strength. These results were supported by the MRM analysis (Table 8), in which time since divergence was the only significant predictor of linguistic distance. The model accounted for 60% of the variation in linguistic diversity across the Ryukyu Islands.

**Table 6:** Simple Mantel correlations between time since divergence, log geographic distance and separation by water for Ryukyuan.

	Time since divergence	Separation by water
Log geographic distance	.824	.365
Separation by water	.210	

**Table 7:** Partial Mantel correlations between linguistic distance, time since divergence, log geographic distance and separation by water for Ryukyuan.

	Linguistic distance			
	r	95% CI		p
Time since divergence	.438	.359	.515	< .001
Log geographic distance	.067	.033	.092	.094
Separation by water	.051	.023	.089	.269
Log geographic * Water	-.025	-.056	.001	.559

**Table 8:** Results for predicting linguistic distance in Ryukyuan using multiple regression over distances matrices.

	Estimate	p
Intercept	0.046	
Time since divergence	1.12·10^−4^	< .001
Log geographic distance	2.15·10^−2^	.092
Separation by water	5.78·10^−2^	.270
Log geographic * Water	-8.08·10^−3^	.563

R^2^ = .603.

The linear mixed model produced results confirming the findings from the Mantel tests (Table 9). Time since divergence and geographic distance were significant predictors of linguistic distance, indicating that the longer ago varieties diverged and the further apart they are, the larger the linguistic distance between them was. The strength of the effect of time since divergence was slightly stronger than the effect of geographic distance. The inclusion of logarithmic geographic distance provided a better model (AIC = -3594.4) than when linear distance was included (AIC = -3525.3). VIF values for the main effects were all < 3.0. As already shown by the Mantel tests above, and reflecting their status as island languages, there was no effect of separation by a body of water for Ryukyuan. Taken together, the effects that time since divergence and geographic distance have on linguistic diversity in Ryukyuan suggest that the patterns of diversity are a reflection of the time since the language varieties diverged—diversity *between* island clusters—but also a reflection of contact between speech communities *within* the island clusters. This is in line with what we predicted for the isolation-by-colonisation situation expected across isolated island clusters that require technology for travel.

**Table 9:** Results for predicting linguistic distance in Ryukyuan using linear mixed effect modeling.

	*β*	*SE*	*t*	*p*
(Intercept)	.010	.063	0.15	
Time-depth	.472	.034	13.75	< .001
Log geographic distance	.282	.035	8.02	< .001
Separation by water	.018	.053	0.33	.739
Log geographic * Water	-.027	.027	0.97	.333

Conditional R^2^ = .694, Marginal R^2^ = .575.

## Discussion

It is clear that geography influences linguistic diversity, just as it influences biological diversity. However, the exact nature of this relationship in the context of languages is still poorly understood. Here we discovered that the geographical configuration of a language area affects the role of two known diversification processes: dispersal and colonisation history. After a cluster analysis based on linguistic distance measures confirmed the legitimacy of Ryukyuan—spoken across isolated island clusters—as a language group distinct from Japanese—spoken across a connected land—we examined the relationship between geographical distance and linguistic diversity, as well as time since divergence and linguistic diversity in these two language areas. As expected, linguistic diversity in both language areas increased with larger geographic distances, and with increased time since speech communities separated for the Ryukyuan area. Importantly, we found that the effect of geographic distance was stronger for Japanese, while the effect of time since divergence was stronger for Ryukyuan—a result of two different processes that have shaped linguistic diversity.

The separation of Japanese varieties has slowly been negated by sustained contact between communities that are geographically close: contact leads to accommodation, which causes varieties to resemble each other more and more as time passes. As a result, we found negative coefficients for time since divergence in our analyses. This effect appears to be strongly driven by the Tokyo variety. The time calibration by Lee and Hasegawa [29] puts it among the oldest clade, but its status as mixed variety (of Eastern and Western Japanese characteristics) that has become the de facto standard has caused it to resemble varieties from both subgroups over time. Interestingly, the relationship between geographic and linguistic distance was linear throughout the entire area, which goes against the general sublinear trend found in other language areas (Bantu, Bulgaria, Germany, US East Coast, the Netherlands, and Norway; see [10]). This indicates that Japanese is a true dialect continuum without any gaps, whereas the sublinear trend found in previously studied language areas could point to the presence of clearly defined, i.e., more isolated, subgroups. It appears that the isolation of subgroups disrupts linguistic continuity in a language area. To demonstrate this, we took the characteristics of the prototypical isolation-by-distance and isolation-by-colonisation patterns (see [12]), and conducted a simulation of linguistic distances between 20 locations across four subgroups. While in this case, isolation-by-adaptation—a scenario in which diversity arises through local adaptation to the natural landscapes [12]; local adaption to a socio-political environment for language—would actually be a better comparison, the contrast with isolation-by-distance remains the same as diversity is not directly related to geographic distance. The simulations indeed showed that increasing the isolation of just one subgroup creates the sublinear trend reported by Nerbonne (see S2 Appendix). In this light, it would be worthwhile to revisit these previously studied language areas to establish whether they differ in the heterogeneity of their linguistic landscapes, which could be an explanation for why linguistic distance appears to reach ceiling at different distances, and moreover, why they vary at all.

In Ryukyuan, the separation of varieties happened a long time ago and has remained largely intact within Ryukyuan due to the difficulties in maintaining contact across isolated islands. Nevertheless, we do find an effect of geographical distance for Ryukyuan—albeit a small effect. This shows that continuum-like characteristics do arise as a result of contact within islands clusters at least for short distances, in line with results from studies that focused on small-scale language areas [55,56]. However, geographic isolation decreases contact beyond the island cluster, which prevents the formation of a continuum across the island chain as a whole. An interesting further step would be to study linguistic diversity in different types of island configurations. The size of islands, as well as the distances between them, affects the potential and frequency of contact between populations, which in turn affects the patterns of overall linguistic diversity, as well as linguistic continuity within a dialect chain.

We also found that overall linguistic diversity was more abundant within Ryukyuan. This goes against what usually happens in population genetics, where a loss of genetic variation usually occurs in a new population as a result of the limited diversity present in its founders [57]. There has been some discussion about whether overall diversity is also reduced in new linguistic communities: suggestions of a decrease in size of the phoneme inventory have been made [58], but this idea is not uncontroversial [59,60]. It is hard to put the specific linguistic distances reported here into broader perspective, as there has been little comparative work across different language/dialect areas. While Nerbonne summarises the general patterns from six language areas [10], each study utilised different units of measurement, providing little opportunity for direct comparison. However, it is not inconceivable that the Ryukyuan language area shows greater overall variation than the ones summarised by Nerbonne, so further work in other island languages is needed to confirm the pattern. Since most fine-grained dialectometric analyses have been applied to land-connected dialect areas, investigating island languages with this approach is an important addition to our knowledge of linguistic diversity. Gavin and Sibanda showed that the *number* of languages per island across the Pacific decreased with each subsequent expansion [11], but they did not examine dialectal variation *within* each language. The methodology applied here creates an opportunity to look at linguistic diversity in a more detailed manner that goes beyond merely counting languages [61].

Finally, the current study used straight-line geographic distances as in population genetics studies, as well as several dialectology studies. An alternative approach would be to measure actual travel time—as has been done for Norway, which is topographically similar to Japan, i.e. mountainous. While travel time between islands will strongly depend on straight line distances over sea, travel across a larger mainland can be hindered by mountain ranges. Modern train distances as a proxy for travel time have been linked to the amount of Standard Japanese vocabulary in dialects across the mainland [62], but the focus lies on two capital locations (Tokyo and Kyoto) as a starting point rather than a location-by-location comparison. Moreover, as land and sea travel have been show to affect the diffusion of linguistic features differently [8], further exploration of historical travel and trade practices—and how they have changed over time—can provide additional insights into the patterns of linguistic diversity we find today.

## Conclusion

To conclude, we have shown that cultural processes—language diversification—are influenced by geography in ways similar to biological processes—species diversification. We examined the role of geographic configuration in diversification and showed that: (I) mainland languages display a typical isolation-by-distance pattern, with gradually increasing diversity over geographic distance, as a result of the higher potential for sustained contact, while (II) island languages display a typical isolation-by-colonisation pattern, where diversity is a reflection of time since divergence, as a result of limited contact due to the geographic isolation of islands. Language variation and change is, of course, influenced by other (historical and socio-political) factors as well, and a more global and multi-dimensional concept of distance—comprising spatial, temporal, and social factors—is needed to help us understand patterns of language diversification. Our results show that the geographical configuration of a language area is one important component of a more comprehensive distance concept to explain language variation and change.

## Supporting information

S1 Supporting information Locations included in the study. (PDF)

S1 Appendix Geography and linguistic diversity in the Japonic language family. (PDF)

S2 Appendix Simulation of geographic and linguistic distances. (PDF)
