# Supplementary material for: The geographical configuration of a language area influences linguistic diversity
Source: PLoS One. 2019 Jun 12;14(6):e0217363. doi: 10.1371/journal.pone.0217363 (PMC6561542; doi:10.1371/journal.pone.0217363)

Japanese locations

1	Aomori	30	Shiga
2	Hirosaki	31	Mie
3	Hachinohe	32	Nara
4	Iwate	33	Kyoto
5	Akita	34	Osaka
6	Miyagi	35	Wakayama
7	Yamagata	36	Hyogo
8	Fukushima	37	Okayama
9	Aizutakada	38	Hiroshima
10	Niigata	39	Yamaguchi
11	Sado	40	Tottori
12	Ibaraki	41	Shimane
13	Tochigi	42	Tokushima
14	Chiba	43	Kagawa
15	Tokyo	44	Kochi
16	Saitama	45	Ehime
17	Gunma	46	Oita
18	Kanagawa	47	Fukuoka
19	Yamanashi	48	Saga
20	Shizuoka	49	Nagasaki
21	Akiyama	50	Nakadori
22	Nagano	51	Fukue
23	Hachijo	52	Miyazaki
24	Toyama	53	Kumamoto
25	Nanao	54	Kagoshima
26	Ishikawa	55	Kamikoshiki
27	Fukui	56	Shimokoshiki
28	Gifu	57	Tanegashima
29	Aichi	58	Yakushima

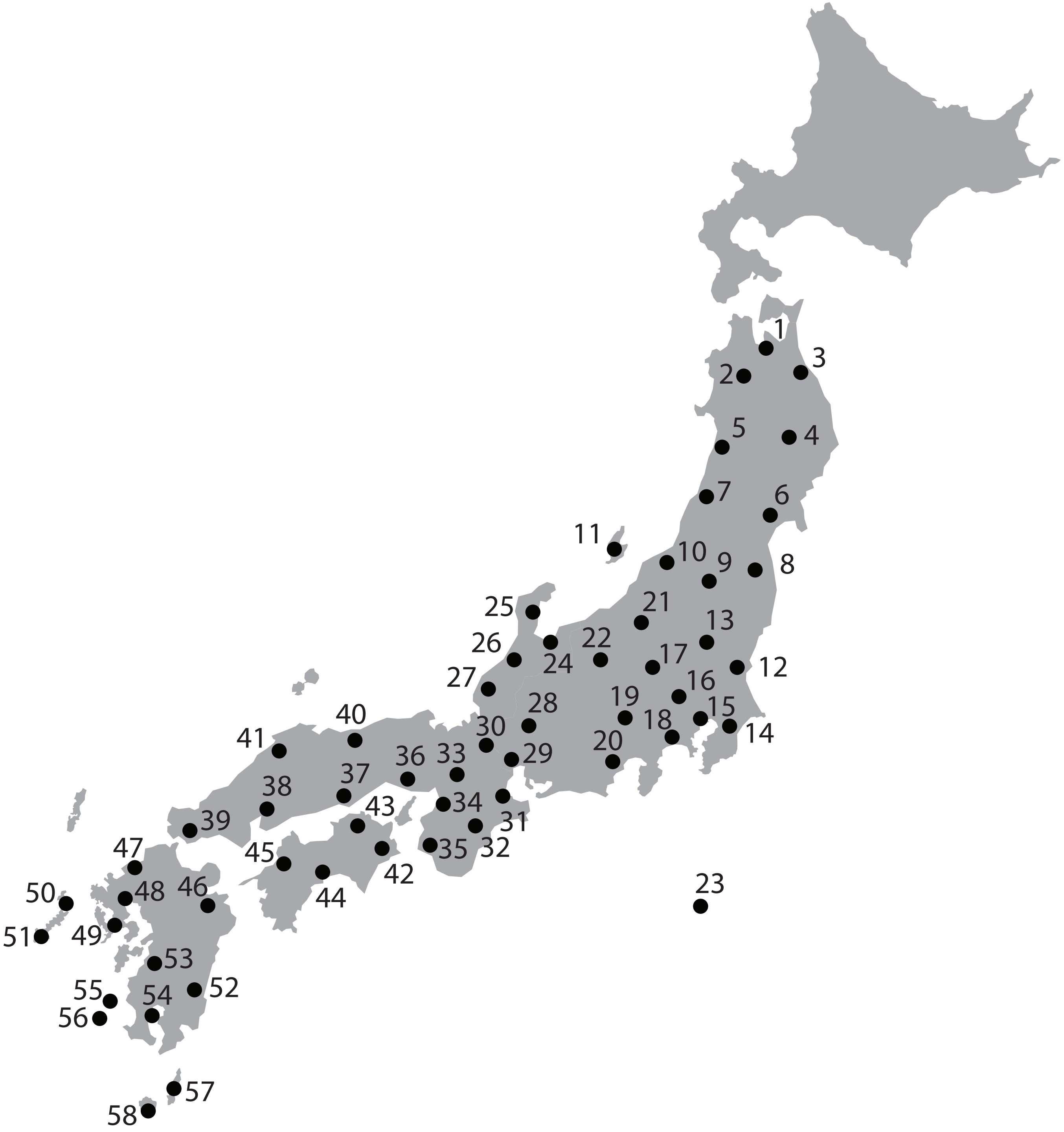

Ryukyuan locations			
59	Kikai	75	Itoman
60	Naze	76	Zamami
61	Sumiyo	77	Kume
62	Koniya	78	Hirara
63	Kakeroma	79	Ikema
64	Tokunoshima	80	Irabu
65	Okinoerabu	81	Tarama
66	Yoron	82	Ishigaki
67	Iheya	83	Taketomi
68	Izena	84	Kohama
69	Nakijin	85	Kuroshima
70	Motobu	86	Aragusuku
71	Nago	87	Hateruma
72	Kin	88	Hatoma
73	Kudaka	89	Iriomote
74	Shuri	90	Yonaguni

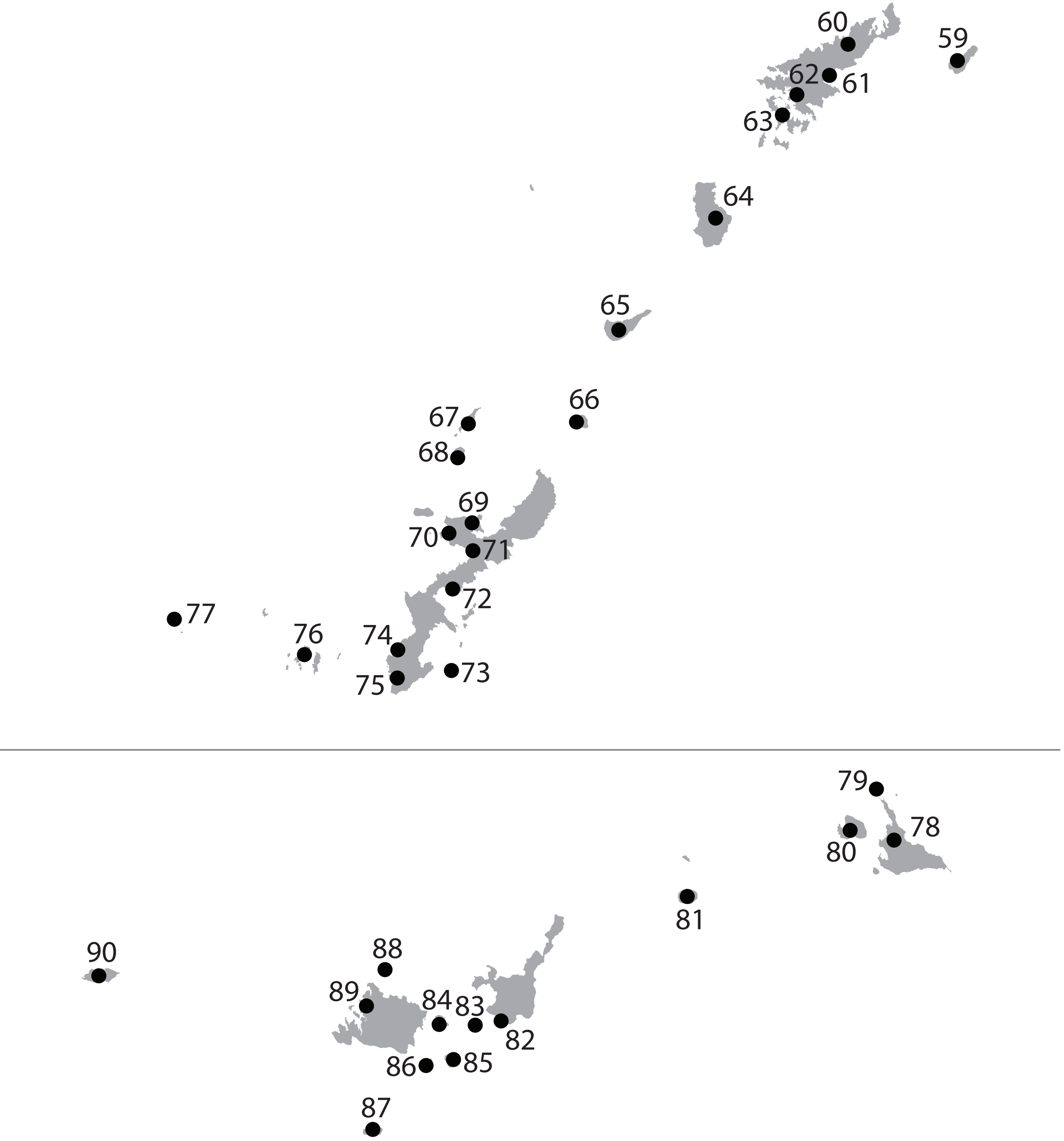

Supplement: S1 Supporting information — (PDF) [file pone.0217363.s001.pdf]
