# Supplementary material for: The geographical configuration of a language area influences linguistic diversity
Source: PLoS One. 2019 Jun 12;14(6):e0217363. doi: 10.1371/journal.pone.0217363 (PMC6561542; doi:10.1371/journal.pone.0217363)
Supplement: S1 Appendix — (PDF) [file pone.0217363.s002.pdf]

The geographical configuration of a language area influences linguistic diversity

John L. A. Huisman, Asifa Majid, Roeland van Hout

S1 Appendix

Geography and linguistic diversity in the Japonic language family

For the Japonic language family as a whole, Mantel tests confirmed that geographic distance, time since divergence, and separation by water are related to each other (Table 1). Partial Mantel tests showed these factors were all positively correlated with linguistic diversity (Table 2). Linguistic distance between language varieties increased as time increases since divergence. Similarly, linguistic distance increases with increased geographic distance—although this effect was slightly weaker. The correlation between linguistic distance and logarithmic geographic distance is not significantly different from its correlation with normal geographic distance, $z = 1.40$, $p = .162$—the latter in fact being numerically stronger; $r = .388$ versus $r = .361$. Linguistic distance also increases when varieties are separated by a body of water. Finally, the negative value for the interaction effect indicated the influence of geographic distance decreased when language varieties were separated by water. These results were supported by the MRM analysis (Table 3), which showed that all factors were significant predictors of linguistic distance, together accounting for 85% of the variation.

Table 1. Simple Mantel correlations between predictors of linguistic diversity for the Japonic language family.

	Time since divergence	Separation by water
Geographic distance	0.729	0.437
Separation by water	0.506	

Table 2. Partial Mantel correlations between linguistic distance, time since divergence, geographic distance and separation by water for the Japonic language family.

	Linguistic distance			
	r	95% CI		p
Time-depth	0.705	0.681	0.727	<.001
Geographic distance	0.388	0.341	0.425	<.001
Separation by water	0.307	0.274	0.351	<.001
Water * Geographic	-0.252	-0.293	-0.215	<.001

Table 3. Results for predicting linguistic distance in the Japonic language family using multiple regression over distances matrices.

	Estimate	p
Intercept	0.111	
Time-depth	$6.46 \cdot 10^{-5}$	<.001
Geographic distance	$1.59 \cdot 10^{-4}$	<.001
Separation by water	$5.96 \cdot 10^{-2}$	<.001
Water * Geographic	$-1.01 \cdot 10^{-4}$	<.001

Note: R^2 = .855.

The findings from the Mantel tests were further confirmed by the results of the mixed model analysis (Table 4), in which time since divergence was again the strongest predictor of linguistic distance, followed by geographic distance, and then separation by water. VIF values for the main effects were all < 2.5. The negative interaction effect confirms a decreased influence of geographic distance for language varieties separated by water. Including normal geographic distance rather than logarithmic distance provided a better model; AIC = -31456.8 versus AIC = -31392.7. Together, this shows that the model is a good reflection of the linguistic situation, accounting for 89% of the variation in linguistic diversity in the Japonic language family.

Table 4. Results for predicting linguistic distance in the Japonic language family using linear mixed effect modeling.

	β	*SE*	*t*	*p*
(Intercept)	.101	.022	4.56	
Time-depth	.592	.006	95.95	<.001
Geographic distance	.423	.007	62.22	<.001
Separation by water	-.124	.009	13.53	<.001
Water*Geographic	-.230	.009	25.66	<.001

Note: conditional R^2 = .890, marginal R^2 = .847.
