# Supplementary material for: The geographical configuration of a language area influences linguistic diversity
Source: PLoS One. 2019 Jun 12;14(6):e0217363. doi: 10.1371/journal.pone.0217363 (PMC6561542; doi:10.1371/journal.pone.0217363)
Supplement: S2 Appendix — (PDF) [file pone.0217363.s003.pdf]

The geographical configuration of a language area influences linguistic diversity

John L. A. Huisman, Asifa Majid, Roeland van Hout

S2 Appendix

Simulation of geographic and linguistic distances

We started out with 20 locations spread cross four subgroups lined up in an archipelago-like fashion (see Fig 1) and calculated geographic distances between them using Euclidean distance. Next, we simulated linguistic distances based on the characteristics of prototypical isolation-by-distance and isolation-by-colonisation patterns as described by Orsini et al. ([1], see Figure 1, p. 5987).

Fig 1. Twenty simulated locations divided into four subgroups.

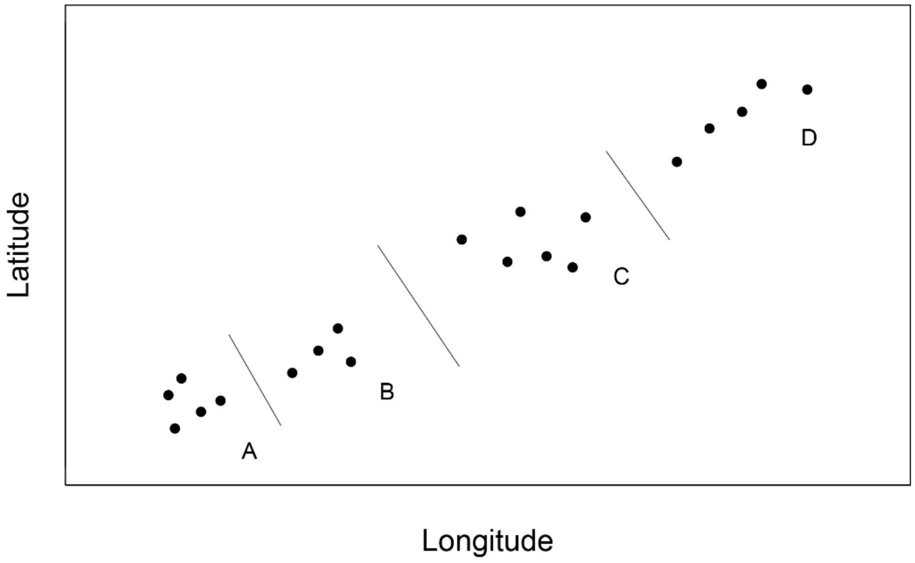

In an isolation-by-distance scenario, there is a direct relationship between geographic and linguistic distance across the whole area. As such, simulated linguistic distance was determined as the geographic distance between two points divided by the maximum geographic distance across the sample, with the addition of some normally distributed

random noise ($M = 0$, $SD = 0.1$). In an isolation-by-colonisation scenario, there is no contact beyond the subgroup and linguistic distance is the result of drift, without any relationship to geographic distance. As such, simulated linguistic distance between locations belonging to different subgroups was determined at a fixed level (d = 0.75), with the addition of normally distributed random noise ($M = 0$, $SD = 0.1$). Simulated linguistic distance within each subgroup was determined following the isolation-by-distance pattern described above. Fig 2 shows the simulation results for the two prototypical scenarios.

Fig 2. Plots of simulated geographic and linguistic distances in isolation-by-distance (left panel) and isolation-by-colonisation (right panel) scenarios.

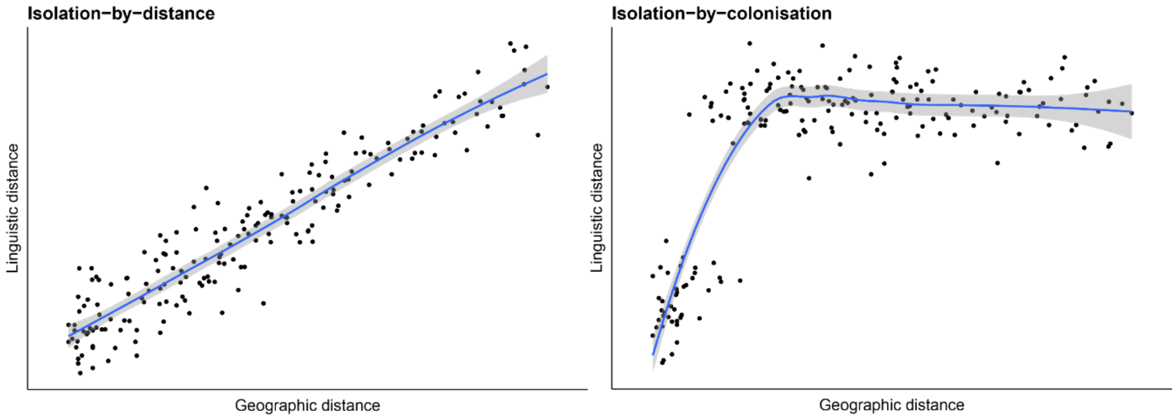

Then, we simulated two scenarios in which one of the peripheral subgroups was isolated from the other three. In that case, simulated linguistic distances between subgroup A (or D) and the other three groups was determined by the isolation-by-colonisation pattern (IBC). The other three subgroups would maintain mutual contact and as such, simulated linguistic distances between them were determined by the isolation-by-distance pattern (IBD). Patterns that determined simulated linguistic distances within and between subgroups are shown in Table 1.

Table 1. Diversification patterns in peripheral isolation scenarios.

Isolation of subgroup A					Isolation of subgroup D				
	A	B	C	D		A	B	C	D
A	*IBD*	**IBC**	**IBC**	**IBC**	A	*IBD*	*IBD*	*IBD*	**IBC**
B		*IBD*	*IBD*	*IBD*	B		*IBD*	*IBD*	**IBC**
C			*IBD*	*IBD*	C			*IBD*	**IBC**
D				*IBD*	D				*IBD*

In addition, we simulated a scenario in which a split between the two "northern" and two "southern" subgroups would result in a situation in which there is only contact between subgroups A and B, and between C and D. The patterns determining simulated linguistic distance within and between subgroups in this scenario are shown in Table 2.

Table 2. Diversification patterns in a north vs. south scenario.

	A	B	C	D
A	*IBD*	*IBD*	**IBC**	**IBC**
B		*IBD*	**IBC**	**IBC**
C			*IBD*	*IBD*
D				*IBD*

The simulations show that linguistic continuity is disrupted in any scenario in which one (Fig 3), several (Fig 4), or all (Fig 2, right panel) subgroups are isolated. The isolation of a subgroups results in high—i.e. higher than expected under an isolation-by-distance pattern—linguistic distances for small geographic distances, which subsequently leads to a sublinear trend as observed in several dialects areas [2].

Fig 3. Plots of simulated geographic and linguistic distances in scenarios where the two peripheral subgroups (A, top; D, bottom) are isolated.

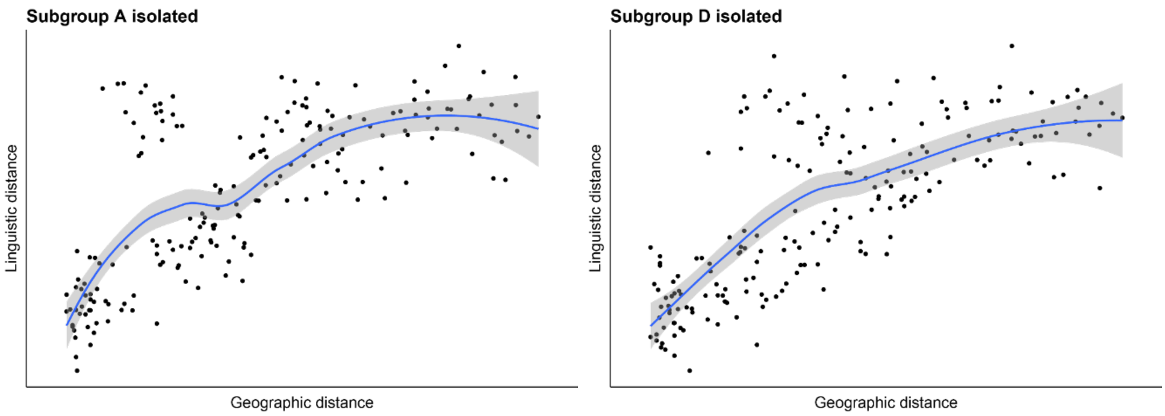

Fig 4. Plot of simulated geographic and linguistic distances in scenarios where the northern (A+B) and southern subgroups (C+D) are isolated.

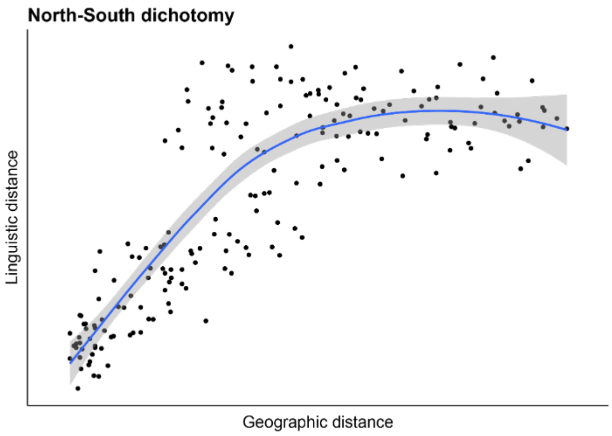

References

1. Orsini L, Vanoverbeke J, Swillen I, Mergeay J, De Meester L. Drivers of population genetic differentiation in the wild: isolation by dispersal limitation, isolation by adaptation and isolation by colonization. Molecular Ecology. 2013;22(24): 5983–5999.
2. Nerbonne J. Measuring the diffusion of linguistic change. Philosophical Transactions of the Royal Society B: Biological Sciences. 2010;365(1559): 3821–3828.
